# RNA World with Inhibitors

**DOI:** 10.3390/e26121012

**Published:** 2024-11-23

**Authors:** Jaroslaw Synak, Agnieszka Rybarczyk, Marta Kasprzak, Jacek Blazewicz

**Affiliations:** 1 Institute of Computing Science, Poznan University of Technology, 60-965 Poznan, Poland; 2 European Center for Bioinformatics and Genomics, 60-965 Poznan, Poland; 3 Institute of Bioorganic Chemistry, Polish Academy of Sciences, 61-704 Poznan, Poland

**Keywords:** RNA world, differential equations, multi-agent systems, chemical kinetics

## Abstract

During the evolution of the RNA World, compartments, which were fragments of space surrounded by a primitive lipid membrane, had to have emerged. These led eventually to the formation of modern cellular membranes. Inside these compartments, another process had to take place—switching from RNA to DNA as a primary storage of genetic information. The latter part needed a handful of enzymes for the DNA to be able to perform its function. A natural question arises, i.e., how the concentration of all vital molecules could have been kept in check without modern cellular mechanisms. The authors propose a theory on how it could have worked during early stages, using only short RNA molecules, which could have emerged spontaneously. The hypothesis was analysed mathematically and tested against different scenarios by using computer simulations.

## 1. Introduction

Originating in the 1960s, the hypothesis of the “RNA World” emphasises the pivotal role of RNA in early life evolution [1,2,3]. It suggests that RNA was essential both to encoding genetic information and to driving enzymatic reactions, marking a significant era in which RNA served uniquely as both a genetic messenger and a catalyst, possessing the capability to replicate itself [4,5,6]. Ongoing research on ribozymes, aptamers, and various cellular RNAs involved in a wide range of biological activities further highlights the versatile role of RNA in the molecular framework of life [7,8,9,10]. This exploration has led to focused efforts to develop and refine RNA polymerase ribozymes, with the aim of assessing the potential for RNA self-replication. Despite the absence of known biological examples of RNA enzymes with RNA-dependent RNA polymerase activity (RNA replicase), substantial advances have been achieved in laboratories through directed evolution methods [10,11,12,13,14]. In particular, the most advanced examples of laboratory-created polymerase ribozymes used in the RNA-catalysed synthesis of complex RNA molecules are evolved descendants of the class I ligase [10,12,14]. Unfortunately, the synthesis fidelity of RNA ribozymes that have been developed experimentally so far, particularly those that involve more intricate RNAs, has been persistently low [15]. Nonetheless, recent efforts suggest that an RNA enzyme with polymerase activity holds potential for replicating and evolving functional RNA strands. While the replication process may still rely on a larger replicase, which limits its applicability, ongoing improvements in RNA polymerase ribozyme fidelity offer promising opportunities to support the evolution of fit RNA-cleaving enzyme variants [16]. Advances like these are encouraging, yet the discovery of an ancient ribozyme with full replication capability remains elusive, and significant challenges lie ahead to demonstrate that RNA alone could sustain an evolving genetic system.

Another key challenge in understanding the origins of life is the lack of error correction mechanisms, which led to frequent mutations in prebiotic replicators. These mutations resulted in a population of quasi-species, consisting of RNA replicases and mutants (parasites defined as RNA molecules lacking polymerase function but capable of being replicated by RNA replicase). Due to high mutation rates, replicase length is limited by replication accuracy. If mutations exceed a threshold, a catastrophe occurs, causing a loss of information. Maynard Smith estimated that the polynucleotide chain size, without enzymes, cannot exceed 100 nucleotides [17], a problem known as the Eigen paradox: RNA must be long enough to function, but being such, it will be quickly overtaken by parasites [18,19,20,21]. Eigen proposed hypercycles as a solution to this problem, but Maynard Smith argued they remain vulnerable to parasites in homogeneous environments [22,23,24].

Additionally, recent advances indicate that RNA did not exist in isolation but coexisted with small peptides and lipids, supporting the compartmentalisation of RNA sequences within prebiotic vesicles (protocells) [25]. These vesicles, formed by a series of self-aggregation processes, could have offered numerous benefits in a world dominated by RNA, particularly supporting complex functions like RNA-catalysed replication reactions [26,27,28]. Compartmentalisation within these vesicles could have enabled the positive feedback essential for evolution by keeping beneficial products close to their catalysts and maintaining higher proximity among closely related molecules compared with those that are more distantly related [29,30,31]. However, membrane-based protocells also face significant challenges, particularly as vesicle boundaries limit access to environmental chemicals, such as charged nucleotides, especially in the absence of developed transport systems. Additionally, the nonenzymatic copying of RNA templates often requires high concentrations of divalent cations for optimal activity, yet these ions also accelerate RNA degradation and destabilise fatty acid vesicles. Chemical compatibility, therefore, becomes a critical concern and may necessitate added components, increasing system complexity [27,28,32]. This is why a wide variety of protocell model systems have been proposed, spanning from entirely inorganic structures to macromolecular aggregates and polymolecular assemblies of amphiphiles [27,33,34,35]. These models include vesicular compartments formed from potentially prebiotic components such as lipidic amphiphiles [27,33], peptides [36], inorganic nanoparticles [37], and polysaccharides [38].

The necessity of compartmentalisation is further underscored by its role in protecting against system crashes due to invasion by parasites [39]. In an unbounded solution, parasitic RNAs, if they serve as superior templates, will proliferate at the cost of functional replicase RNAs, leading to population collapse [40,41]. In a population of small compartments, the rise of a parasitic sequence in one compartment will not affect the overall population, even if it signifies disaster for the descendants of that compartment. Empirical evidence suggests that even temporary compartmentalisation is sufficient to prevent parasite-triggered extinction [27,42,43,44].

To date, many modelling methods have been proposed to study prebiotic systems, with theoretical modelling and computer simulation emerging as the most effective approaches to addressing issues related to evolutionary dynamics [45]. Among these, mathematical models, such as the minimal replicator network, which includes one replicase and one parasite species (shortly referred to as the RP model), have attracted significant attention [46]. At the same time, various computational strategies have been used, including probabilistic models [47], discrete models such as cellular automata [46,48], multi-agent-based approaches (MAS) [40,41,49], artificial chemistry [50], and protocell simulation using molecular dynamics models [47]. Prebiotic reactions have also been studied in detail with algorithms based on quantum dynamics [51].

This paper focuses on two particular modelling strategies, multi-agent systems (MASs) and ordinary differential equations (ODEs), to explore how concentrations of vital molecules might have been regulated in early stages without modern cellular mechanisms, using short spontaneously emerged RNA molecules. These short RNAs can bind to larger ones via Watson–Crick pairing, blocking replication when attached, effectively acting as “inhibitors”. On the contrary, “anti-inhibitors” can bind and neutralise these inhibitors, ensuring continued molecular interactions. All the reactions described are fully reversible.

The proposed model aligns with the findings of previous studies, which have highlighted the importance of the tag mechanism in enhancing the expansion and distribution of polymerase ribozymes in both naked and cellular environments [31,45], which remains relevant even in more complex systems that include multiple ribozymes [31]. The “tag” in this context refers to a short specific sequence, often located at the 5^′^ end of RNA.

Additionally, the model presented here parallels the concept explored in earlier experiments with a hammerhead ribozyme [52]. The key distinction, however, is that in the previous study, the control mechanisms relied solely on the concentration of specific molecules. In contrast, the current model introduces the possibility of regulation through inhibitor–anti-inhibitor interactions, offering a more sophisticated layer of control.

Moreover, we carried out comprehensive simulation tests for both models. In the end, we compared them to discern the universal laws and norms that control their dynamics and evolution. These simulations aligned with those of previous research in which we demonstrated that tackling the description and analysis of the RP system from various points of view (microscopic and macroscopic) produces congruent outcomes [49].

## 2. Materials and Methods

### 2.1. Biological Model

The RNA World hypothesis posits that RNA molecules could have gained the ability to catalyse replication, which allowed stable populations of RNA molecules to arise [4]. Eventually, these had to be enclosed in membranes, creating protocells, which, after aeons, of evolution turned into modern cells [27]. According to the RNA World hypothesis, RNA molecules were the first enzymes, so inside a protocell, they could perform different functions, resulting in several groups of specialised molecules. Modern cells require the concentration of all their enzymes to be tightly controlled, so it is not beyond the realm of possibility that millions of years ago, some mechanisms maintaining the proper concentration of each type of RNA were necessary. While unrestricted replication could have been beneficial during the early stages of the RNA World, the moment multiple different enzymes emerged and began to interact, the uncontrolled proliferation of any one of them could have starved the rest and destroyed the metabolism. Thus, protocells with said mechanisms in place could have had a huge advantage, since they were more resilient, as any (random or caused by the external environment) changes to RNA concentrations inside them could have been corrected. The first such mechanisms had to be simple, so that they could arise by chance. The one proposed in this article and further described below fulfils this condition due to its relative simplicity. Furthermore, it does not rely on any new molecule, as it is based purely on RNA. It should be mentioned that a very similar idea to the one described below has been proposed several years ago and tested with the use of a hammerhead ribozyme, yielding promising results. The main difference is that in the aforementioned work the activity of control mechanisms depended solely on the concentration of proper molecules, while in the model presented in this article, it can happen also due to inhibitor–anti-inhibitor pairing [52].

The model proposed in this publication is based on the following assumptions:

1. One of the steps of RNA World emergence was the transition β→γ, when ribonucleotides started to form longer chains [40]. Apart from functional molecules, which, during the next stage (γ→δ), self-organised into more complicated systems, a lot of shorter, non-functional RNAs should have also emerged. Their creation could be explained by primitive, imperfect polymerisation, which can easily stop ahead of time, when anything goes wrong.
2. Short RNA molecules can bind to larger ones based on Watson–Crick pairing.
3. An RNA molecule with a shorter RNA attached cannot be replicated, since there is no access to its sequence. If the molecule is also an enzyme, then it cannot fold into its proper structure and is rendered non-functional. Molecules with the ability to block longer RNAs like this are called “inhibitors” in this model. While oligonucleotides can also act as replication intermediates, only their inhibiting activity is investigated in this article [52].
4. Inhibitors can bind with their complementary molecules, which prevents them from blocking anything; the latter group is called “anti-inhibitors”.
5. The reactions described above are fully reversible. They can go in both directions.

This approach can be corroborated to some extent by the following facts:

1. Advanced mechanisms controlling cell metabolism had to evolve from something much more primitive, a system which could have emerged by chance and function in early lifeforms [52].
2. In the present, there are sequence fragments called riboswitches, which sometimes occur in the mRNA of modern cells. Riboswitches can regulate the function of mRNA by binding to various molecules. Their presence and the way they function may suggest that they are remnants of much older mechanisms existing back in the RNA World [53].

**Figure 1:**
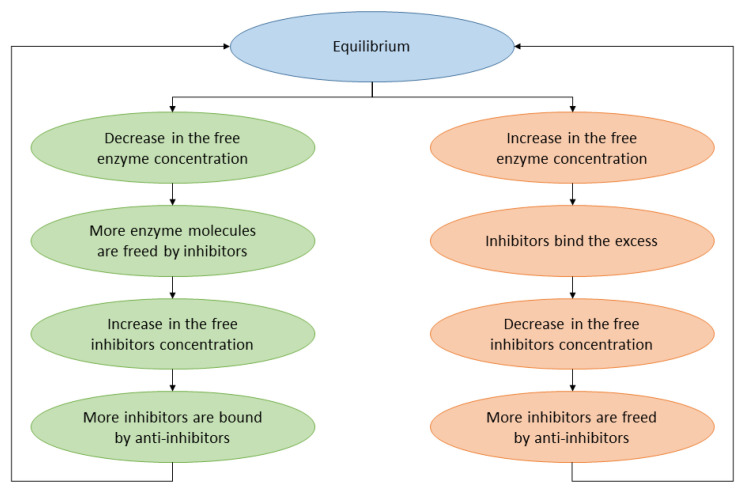
The feedback loop (drawn in two variants) in the system ensures that the concentration of RNA enzymes and inhibitors remains constant.

The general schema of the model is presented in Figure 1.

### 2.2. Mathematical Model

To better understand the properties of such a system, a simpler case is considered first with anti-inhibitors excluded. Then, anti-inhibitors are introduced to observe what difference they can make. The main tools used to describe the model are differential equations, which are extensively analysed in the following subsections.

#### 2.2.1. Agents and Inhibitors

In this scenario, there are only two types of molecules: agents (active molecules denoted by *A*) and inhibitors (*X*). They can form inactive complexes in a reversible reaction:

$$A+X\overset{k_{1}}{\underset{k_{2}}{⇌}}AX$$

All reactions are assumed to be in accordance with the law of mass action. The (positive) constants $k_{1}$ and $k_{2}$ in the reaction above are its rate constants. By modelling the molar concentrations [A], [X], and [AX] as functions of time, one can instantly infer three differential equations describing their evolution:

(1) $$\frac{d[A]}{dt}=k_{2}\left[AX\right]-k_{1}\left[A\right]\left[X\right]$$

(2) $$\frac{d[X]}{dt}=\frac{d[A]}{dt}$$

(3) $$\frac{d[AX]}{dt}=-\frac{d[A]}{dt}$$

If the right side of the Equation (1) is positive, the concentrations [A] and [X] will be increasing, while [AX] will decrease, so the value of $k_{2}\left[AX\right]-k_{1}\left[A\right]\left[X\right]$ will also drop. If the right side of Equation (1) is negative, $k_{2}\left[AX\right]-k_{1}\left[A\right]\left[X\right]$ will be increasing, meaning that this value always tends to 0. In other words, the system tends towards an equilibrium, which can be described by the equation below:

(4) $$\begin{array}{c} k_{2}\left[AX\right]-k_{1}\left[A\right]\left[X\right]=0 \end{array}$$

(5) $$\begin{array}{c} \frac{k_{2}}{k_{1}}\left[AX\right]=\left[A\right]\left[X\right] \end{array}$$

By denoting the concentrations in the equilibrium by $e_{A}$, $e_{X}$, and $e_{AX}$ and introducing the equilibrium constant $K=\frac{k_{2}}{k_{1}}$, one obtains the following equation:

(6) $$Ke_{AX}=e_{A}e_{X}$$

It is convenient to introduce two additional positive constants—the total concentrations of *A* and *X* in the solution:

(7) $$c_{A}=\left[A\right]+\left[AX\right]=e_{A}+e_{AX}$$

(8) $$c_{X}=\left[X\right]+\left[AX\right]=e_{X}+e_{AX}$$

For the purpose of this analysis, the equations above can be written as

(9) $$e_{AX}=c_{A}-e_{A}$$

(10) $$e_{X}=c_{X}-c_{A}+e_{A}$$

By using the relations above and denoting $e_{A}$ by *x*, we can rewrite Equation (6):

(11) $$\begin{array}{c} K\left(c_{A}-x\right)=x\left(c_{X}-c_{A}+x\right) \end{array}$$

(12) $$\begin{array}{c} x^{2}+\left(c_{X}-c_{A}+K\right)x-Kc_{A}=0 \end{array}$$

In order to check for potential solutions, it is convenient to define the left side of the equation above as the value of a real function f(y) for y=x:

(13) $$f\left(y\right)=y^{2}+\left(c_{X}-c_{A}+K\right)y-Kc_{A}$$

Let us calculate two chosen points:

(14) $$\begin{array}{c} f\left(0\right)=-Kc_{A}<0 \end{array}$$

(15) $$\begin{array}{c} f\left(c_{A}\right)=c_{X}c_{A}>0 \end{array}$$

The function changes its sign between 0 and $c_{A}$, meaning there is an odd number of roots in this range. As a second-degree polynomial, it can have two roots at most, so there is always exactly one root in the aforementioned range. The coefficient of $y^{2}$ is positive, so f(y) is always positive for sufficiently big arguments and there is no root $y\geq c_{A}$. The roots of f(y) can be calculated with the quadratic formula

(16) $$\Delta ={\left(c_{X}-c_{A}+K\right)}^{2}+4Kc_{A}>0$$

because the biggest of the roots is the one being searched for:

(17) $$x=\frac{-\left(c_{X}-c_{A}+K\right)+\sqrt{\Delta }}{2}$$

By treating *x* as a function of constants $c_{A}$, $c_{X}$, and *K*, we can define the capacity of a solution:

(18) $$\beta =1/\frac{\partial x}{\partial c_{A}}$$

This value can be a measure of how well the system can counter any changes in concentrations in order to keep them within acceptable levels (how sensitive *x* is to any changes in $c_{A}$). The higher the capacity, the better it performs. One of the ways to analyse the formula above is to use Equation (17), but the calculations quickly get complicated and make it hard to see the most important properties of β. One can approach the problem from another angle and start by differentiating Equation (12) with respect to $c_{A}$:

(19) $$\begin{array}{c} 2x\frac{\partial x}{\partial c_{A}}+\left(c_{X}-c_{A}+K\right)\frac{\partial x}{\partial c_{A}}-x-K=0 \end{array}$$

(20) $$\begin{array}{c} \beta =1/\frac{\partial x}{\partial c_{A}}=\frac{2x+c_{X}-c_{A}+K}{x+K} \end{array}$$

We multiply the numerator and the denominator by *x*:

(21) $$\beta =\frac{2x^{2}+c_{X}x-c_{A}x+Kx}{x^{2}+Kx}$$

Equation (12) can be written as

(22) $$c_{X}x=-x^{2}+\left(c_{A}-K\right)x+Kc_{A}$$

By substituting this into Equation (21), we obtain

(23) $$\beta =\frac{x^{2}+Kc_{A}}{x^{2}+Kx}$$

For fixed *K* and $c_{A}$, all values of *x* between 0 and $c_{A}$ are possible, so one only has to choose a correct value of $c_{X}$. This can be proven with Equation (22):

(24) $$\begin{array}{c} c_{X}x=-x^{2}+\left(c_{A}-K\right)x+Kc_{A}=\left(c_{A}-x\right)\left(x+K\right) \end{array}$$

(25) $$\begin{array}{c} c_{X}=\frac{\left(c_{A}-x\right)\left(x+K\right)}{x}>0 \end{array}$$

The parameter $c_{X}$ calculated like this is always positive, so it fulfils the conditions.

Returning to Equation (23), we know that $x<c_{A}$, so the numerator is bigger than the denominator, and this gives 1 as the lower bound for β. The upper bound is equal to $c_{A}/x$, which can be easily proven:

(26) $$\beta =\frac{c_{A}}{x}\frac{x\frac{x}{c_{A}}+K}{x+K}$$

Since $x/c_{A}<1$, the numerator of the second factor is smaller than its denominator, so $\beta <c_{A}/x$, which was to be demonstrated. To sum up the results,

(27) $$1<\beta <\frac{c_{A}}{x}$$

It means that the biggest values of β are possible if $x<<c_{A}$, which can be shown by taking the appropriate limit of expression (23) for fixed *K* and $c_{A}$:

(28) $$\lim_{x\to 0}\beta =\lim_{x\to 0}\frac{x^{2}+Kc_{A}}{x^{2}+Kx}=\infty$$

Any small value of *x* is achievable, which has been proven (it is enough to choose $c_{X}$ accordingly).

#### 2.2.2. The Model with Anti-Inhibitors Included

Here, we assume three kinds of molecules present in the solution: agents (*A*), inhibitors (*X*), and anti-inhibitors (*Y*). Two reversible reactions can occur:

(29) $$A+X\overset{k_{1}}{\underset{k_{2}}{⇌}}AX$$

(30) $$X+Y\overset{k_{3}}{\underset{k_{4}}{⇌}}XY$$

Just like in the previous section, it is straightforward to write differential equations modelling the evolution of the system:

(31) $$\frac{d[A]}{dt}=k_{2}\left[AX\right]-k_{1}\left[A\right]\left[X\right]$$

(32) $$\frac{d[AX]}{dt}=-\frac{d[A]}{dt}=k_{1}\left[A\right]\left[X\right]-k_{2}\left[AX\right]$$

(33) $$\frac{d[Y]}{dt}=k_{4}\left[XY\right]-k_{3}\left[X\right]\left[Y\right]$$

(34) $$\frac{d[XY]}{dt}=-\frac{d[Y]}{dt}=k_{3}\left[X\right]\left[Y\right]-k_{4}\left[XY\right]$$

(35) $$\frac{d[X]}{dt}=k_{2}\left[AX\right]-k_{1}\left[A\right]\left[X\right]+k_{4}\left[XY\right]-k_{3}\left[X\right]\left[Y\right]$$

Based on the simpler version of the model, here, it is also convenient to introduce positive constants which represent the total concentrations of three basic molecules:

(36) $$c_{A}=[A]+[AX]$$

(37) $$c_{X}=[X]+[AX]+[XY]$$

(38) $$c_{Y}=[Y]+[XY]$$

Based on Equations (31), (33), and (35), one can write the equilibrium equations

(39) $$\begin{array}{c} K[AX]=[A][X] \end{array}$$

(40) $$\begin{array}{c} k[XY]=[X][Y] \end{array}$$

where $K=\frac{k_{2}}{k_{1}}$ and $k=\frac{k_{4}}{k_{3}}$.

##### Stability

The system of differential Equations (31)–(35) seems complex at first glance, and it is not entirely clear if the mixture tends to any equilibrium and if there always exists a stable one. In this subsection, it will be assumed it exists for every set of parameters (which will be shown to be true in the next subsection) in order to prove that the system tends to it.

The state of the solution is described by using five variables: [A], [X], [Y], [AX], and [XY]. The first thing to check is if all of them remain nonnegative at all times, which is required by their definition. As functions of time, they are differentiable, so they have to be continuous. Hence, to reach any negative value, 0 has to be reached before. By taking into account the equations defining constants $c_{A}$, $c_{X}$, and $c_{Y}$, it is easy to notice that the derivative with respect to time of any concentration of an uncomplexed molecule ([A], [X], and [Y]) equal to 0 is positive. Indeed, negative components in each of the differential equations are directly proportional to the value of the variable itself, meaning that if the variable is 0, the right side cannot be negative. Moreover, Equations (36)–(38) mean that the positive components in each equation have a sum greater than 0. To summarise, if [A], [X] or [Y] reaches 0, its corresponding derivative is positive, so the value will not become negative at any point. There is another important conclusion which should be mentioned. Since, in the equilibrium, the derivatives should be all 0, it means that in such case, the concentrations [A], [X], and [Y] have to be positive.

The proof for [AX] and [XY] is very similar, except for one special case where [X]=0. First, let us assume that [AX] became negative at some point in time $t_{0}$ (i.e., [AX]=0 at $t_{0}$). It could happen only when [X]=0. Since the derivative of [X] is continuous and had to be positive at $t_{0}$, it means that for some short time after $t_{0}$, [X] was increasing (it quickly became positive then). Since the function [AX] is continuous, when it became negative, it had to remain so for some time after $t_{0}$. Let us define a small real number ϵ such that in the time range $\left(t_{0};t_{0}+\epsilon \right]$, [AX] was negative and [X] positive (and increasing). [A] had to be positive during that time, which is a consequence of Equation (36). It is obvious now from Equation (32) that the derivative of [AX] had to be positive for $t\in (t_{0};t_{0}+\epsilon ]$. However, the mean value theorem states that since [AX] was 0 at $t_{0}$ and negative at $t_{0}+\epsilon$, its derivative had to be negative at some point—a contradiction. The reasoning for [XY] is analogous.

Now, it is proven that all variables in this model (given that the initial state was defined correctly) will always remain within acceptable ranges. However, it still does not explain what is the direction of system evolution. The first step in analysing this is choosing the smallest set of variables that uniquely describe the solution’s state. This can be performed by slightly rewriting the equations for $c_{A}$, $c_{X}$, and $c_{Y}$:

(41) $$\left[AX\right]=c_{A}-\left[A\right]$$

(42) $$\left[XY\right]=c_{Y}-\left[Y\right]$$

(43) $$\left[X\right]=c_{X}-c_{A}+\left[A\right]-c_{Y}+\left[Y\right]$$

It is clear now that all variables can be derived from [A] and [Y], so these two concentrations are enough to fully describe the state of the system. At this point, it is convenient to introduce new variables:

(44) $$\begin{array}{c} x+x_{0}=\left[A\right] \end{array}$$

(45) $$\begin{array}{c} y+y_{0}=\left[Y\right] \end{array}$$

where $x_{0}$ and $y_{0}$ are defined as concentrations of *A* and *Y*, respectively, in a chosen equilibrium state (its existence is assumed at the beginning of the subsection).

The substitution of expressions (41)–(45) into differential Equations (31) and (33) gives us

(46) $$\dot{x}=k_{2}\left(c_{A}-x_{0}\right)-k_{1}x_{0}\left(c_{X}-c_{A}+x_{0}-c_{Y}+y_{0}\right)-k_{2}x-k_{1}x_{0}\left(x+y\right)-k_{1}x\left[X\right]$$

(47) $$\dot{y}=k_{4}\left(c_{Y}-y_{0}\right)-k_{3}y_{0}\left(c_{X}-c_{A}+x_{0}-c_{Y}+y_{0}\right)-k_{4}y-k_{3}y_{0}\left(x+y\right)-k_{3}y\left[X\right]$$

[X] has been left in two places to make the equations slightly shorter to write. They can be further simplified. Let us use Equations (39) and (40) with the substitution as in (41)–(43) in the equilibrium state:

(48) $$\begin{array}{c} k_{2}\left(c_{A}-x_{0}\right)=k_{1}x_{0}\left(c_{X}-c_{A}+x_{0}-c_{Y}+y_{0}\right) \end{array}$$

(49) $$\begin{array}{c} k_{4}\left(c_{Y}-y_{0}\right)=k_{3}y_{0}\left(c_{X}-c_{A}+x_{0}-c_{Y}+y_{0}\right) \end{array}$$

These can be used to eliminate the first two components in each of the formulas for $\dot{x}$ and $\dot{y}$:

(50) $$\dot{x}=-k_{2}x-k_{1}x_{0}\left(x+y\right)-k_{1}x\left[X\right]$$

(51) $$\dot{y}=-k_{4}y-k_{3}y_{0}\left(x+y\right)-k_{3}y\left[X\right]$$

To prove that the point of equilibrium (x,y)=(0,0) is stable, the Lyapunov method can be used [54]. The first step is to define a function of the system state:

(52) $$V\left(x,y\right)=k_{3}y_{0}x^{2}+k_{1}x_{0}y^{2}$$

If the function *V* satisfies all the necessary conditions, it will be a Lyapunov function, the existence of which automatically proves the equilibrium’s stability. Since $x_{0},y_{0}>0$ (which has already been proven), the function is positive everywhere except the equilibrium point (0,0), so the latter is a local minimum. The gradient of *V* at every point can be easily calculated:

(53) $$\frac{\partial V}{\partial x}=2k_{3}y_{0}\;x$$

(54) $$\frac{\partial V}{\partial y}=2k_{1}x_{0}y$$

The total derivative of *V* with respect to time can be written as

(55) $$\frac{dV}{dt}=\frac{\partial V}{\partial x}\dot{x}+\frac{\partial V}{\partial y}\dot{y}$$

We then substitute the partial derivatives, $\dot{x}$ and $\dot{y}$:

(56) $$\frac{dV}{dt}=-2k_{2}k_{3}y_{0}x^{2}-2k_{1}k_{4}x_{0}y^{2}-2k_{1}k_{3}x_{0}y_{0}{\left(x+y\right)}^{2}-2k_{1}k_{3}\left(y_{0}x^{2}+x_{0}y^{2}\right)\left[X\right]$$

All the components are nonpositive and the value of the total derivative is negative everywhere except the equilibrium point. In other words, the value of *V* always decreases with time until it reaches its local minimum—the point of equilibrium (0,0). There is also another important conclusion—if the point of equilibrium exists, it is unique.

##### Determining the Equilibrium Point

In the previous subsection, the existence of an equilibrium point was merely assumed; now, it will be actually proven. In order not to introduce several new symbols unnecessarily, the concentrations [A], [X], [Y], [AX], and [AY] will not be treated as functions of time anymore, but rather as concentrations in the state of equilibrium. As a first step, Equation (39) with the substitution of (41) has the form

(57) $$K(c_{A}-\left[A\right])=\left[A\right]\left[X\right]$$

For convenience, one can denote [A]=x:

(58) $$\left[X\right]=K\frac{c_{A}-x}{x}$$

By substituting Formulas (41) and (42) in Equation (37),

(59) $$\left[Y\right]=\left[X\right]+c_{A}-x-c_{X}+c_{Y}$$

By using Formulas (42) and (59), we can write Equation (40) as

(60) $$k\left(c_{X}+x-\left[X\right]-c_{A}\right)=\left[X\right]\left(\left[X\right]+c_{A}-x-c_{X}+c_{Y}\right)$$

After multiplying both sides by $x^{2}$,

(61) $$kx\left(c_{X}x+x^{2}-\left[X\right]x-c_{A}x\right)=\left[X\right]x\left(\left[X\right]x+c_{A}x-x^{2}-c_{X}x+c_{Y}x\right)$$

By substituting Formula (58) and rearranging, we obtain

(62) $$\left(k-K\right)x^{3}+\left[\left(k-K\right)\left(c_{X}+K-c_{A}\right)+K\left(c_{A}+c_{Y}\right)\right]x^{2}+Kc_{A}\left(2K-k-c_{A}+c_{X}-c_{Y}\right)x-K^{2}c_{A}^{2}=0$$

Because the polynomial above is quite long, it is convenient to denote its coefficients with letters:

(63) a=k−K

(64) $$b=\left(k-K\right)\left(c_{X}+K-c_{A}\right)+K\left(c_{A}+c_{Y}\right)$$

(65) $$c=Kc_{A}\left(2K-k-c_{A}+c_{X}-c_{Y}\right)$$

(66) $$d=-K^{2}c_{A}^{2}$$

We denote the polynomial by L(x); naturally, the equilibrium state corresponds to a root within the range $\left(0,c_{A}\right)$, so it is necessary to check for its existence.

(67) $$L\left(0\right)=-K^{2}c_{A}^{2}<0$$

(68) $$L\left(c_{A}\right)=kc_{X}c_{A}^{2}>0$$

Since L(x) is continuous, according to the intermediate value theorem, there should be at least one root within the range $\left(0,c_{A}\right)$. In the previous subsection, it has been proven that there can be at most one equilibrium state, so the function L(x) always has exactly one root within this range.

One thing has been assumed without a proof, that is, every root within the range $\left(0,c_{A}\right)$ corresponds to a correct equilibrium state. It can be proven as follows: The question of whether a root corresponds to a proper equilibrium is the question of whether it does not violate the constraint discussed in the previous subsections (no concentration can be negative). The polynomial L(x) has been derived from Equations (36)–(40); in fact, it is just Equation (40) after some substitutions, and it allows us to calculate [A]. The remaining formulas can be rewritten to obtain [AX], [X], [Y], and [XY] (Equations (41), (42), (58), and (59)). Now, the signs of all concentrations can be analysed. [A] is positive and lower than $c_{A}$, since it has to lie within the range $\left(0,c_{A}\right)$, which means that [X] and [AX] also have to be positive. The concentrations [Y] and [XY] can be both negative or both positive in order to fulfil Equation (40). However, Equation (38) allows only for the second option.

##### System Capacity

Just like in the simpler version of the model, the system capacity can be defined. The parameter x=[A] (the concentration of *A* in the state of equilibrium) can be treated as a function of five constants: *K*, *k*, $c_{A}$, $c_{X}$, and $c_{Y}$. This approach is justified because their values uniquely determine the value of *x*. The capacity can be defined with the same formula as previously:

(69) $$\beta =1/\frac{\partial x}{\partial c_{A}}$$

The capacity represents how a small change of $c_{A}$ influences *x*, in other words, how the system reacts if some portion of *A* is added or removed. Since writing the exact formula for *x* would be very tedious, it is easier to calculate β by differentiating Equation (62) with respect to $c_{A}$:

(70) $$\left(3ax^{2}+2bx+c\right)\frac{\partial x}{\partial c_{A}}-ex^{2}-fx-g=0$$

where

(71) $$e=-\frac{\partial b}{\partial c_{A}}=k-2K$$

(72) $$f=-\frac{\partial c}{\partial c_{A}}=-K\left(2K-k-2c_{A}+c_{X}-c_{Y}\right)$$

(73) $$g=-\frac{\partial d}{\partial c_{A}}=2K^{2}c_{A}$$

So, β can be written as

(74) $$\beta =\frac{3ax^{2}+2bx+c}{ex^{2}+fx+g}$$

By multiplying the numerator and the denominator by $c_{A}x$,

(75) $$\beta =\frac{c_{A}}{x}\frac{3ax^{3}+2bx^{2}+cx}{c_{A}ex^{2}+c_{A}fx+c_{A}g}$$

The variable *x* is a solution of the equation L(x)=0, so L(x) can be safely added to the denominator and subtracted from the numerator:

(76) $$\beta =\frac{c_{A}}{x}\frac{2ax^{3}+bx^{2}-d}{ax^{3}+\left(b+c_{A}e\right)x^{2}+\left(c+c_{A}f\right)x+\left(d+c_{A}g\right)}$$

It is easy to show that

(77) $$b+c_{A}e=\left(k-K\right)\left(c_{X}+K\right)+Kc_{Y}$$

(78) $$c+c_{A}f=Kc_{A}^{2}$$

(79) $$d+c_{A}g=K^{2}c_{A}^{2}$$

So, β can be rewritten as

(80) $$\beta =\frac{c_{A}}{x}\frac{2ax^{3}+bx^{2}+K^{2}c_{A}^{2}}{ax^{3}+\left(b+c_{A}e\right)x^{2}+\left(c+c_{A}f\right)x+K^{2}c_{A}^{2}}$$

Now, it can be proven that the second factor is always positive and less than 1. First, one can define a polynomial being the denominator subtracted from the numerator:

(81) $$G\left(x\right)=ax^{3}-c_{A}ex^{2}-Kc_{A}^{2}x=\left(k-K\right)x^{3}+c_{A}\left(2K-k\right)x^{2}-Kc_{A}^{2}x$$

It can be factored as

(82) $$G\left(x\right)=-x\left(c_{A}-x\right)\left[kx+K\left(c_{A}-x\right)\right]$$

The value of *x* lies within the range $\left(0,c_{A}\right)$, so G(x)<0. It means that in Equation (80), the numerator is always smaller than the denominator. Moreover, the numerator in Equation (74) is the derivative of L(x) with respect to *x*, and since, at *x*, the sign of L(x) changes from minus to plus, the derivative has to be nonnegative at this point. It should be mentioned that L(x) was subtracted from the numerator along the way, but *x* is its root, so L(x)=0. One can easily conclude that

(83) $$0<\beta <\frac{c_{A}}{x}$$

##### Calculating $c_{X}$ and $c_{Y}$ for Given $c_{A}$, *K*, *k*, and [A]

Looking at the problem from a more practical point of view, one can ask if it is possible to design the system to maintain any given value of *A* by manipulating $c_{X}$ and $c_{Y}$. It can be performed by rewriting Equation (62):

(84) $$Sc_{X}-Tc_{Y}=U$$

where

(85) $$S=x[kx+K\left(c_{A}-x\right)]$$

(86) $$T=Kx(c_{A}-x)$$

(87) $$U=\left(K-k\right)x^{3}+\left[\left(K-k\right)\left(K-c_{A}\right)-Kc_{A}\right]x^{2}-Kc_{A}\left(2K-k-c_{A}\right)x+K^{2}c_{A}^{2}$$

It is obvious that both *S* and *T* are positive. By taking correct values of $c_{X}$ and $c_{Y}$, it is always possible to fulfil Equation (84), which means that for any value [A], the system can maintain it, if the total amounts of *X* and *Y* are chosen properly.

### 2.3. Computer Simulation Algorithms

The system described in the previous subsections was modelled with different approaches according to these scenarios:

1. Solving the differential equations numerically by using spreadsheets;
2. Testing how well the model performs against a predefined function governing changes in [A] by using a program in C++;
3. Simulating the system with self-replicating RNAs (replicases) by using a simulator implemented in C++;
4. Simulating the system with replicases and parasites by using a multi-agent approach.

The implementation in scenarios 1–4, along with the results obtained, is described.

### 2.4. Evolutionary Role of Parameters

The constants $k_{1}$, $k_{2}$, $k_{3}$, and $k_{4}$ represent the chemical reaction rates, which determine the equilibrium and, which is equally important, how quick the system can respond to any changes in concentrations. The inhibitors have to be fast enough to counter any critical concentration disturbance in time, so the constants $k_{i}$ should in theory evolve towards higher values. However, over-reactive molecules can be detrimental, for example, if the reactions described in this article use energy (increase entropy). In that case, inhibitors would put more strain on the protocell than necessary for their function, effectively weakening it.

Another extremely important variable is β, which is a function of constants $k_{i}$ and inhibitors–anti-inhibitors concentrations. As described above, it determines the system’s ability to return to its equilibrium after major changes in the regulated enzyme’s concentration. In theory, the concentration should regain its original values after a disturbance (given enough time); nevertheless, nothing is perfect, even though the new value should be close to the original one, they will never be exactly equal. From this perspective, one can define β as a measure of how imperfect the system is, with infinity representing an unachievable ideal. However, from the biological point of view, too high a β can also be detrimental. Small fluctuations in enzymes’ concentrations can be beneficial as a way to better adapt to the current external situation.

## 3. Results and Discussion

Several computer simulations were performed by the authors in order to test various aspects of the model described. The limitations of inhibitors were checked by analytical methods in one of the previous sections. However, this does not give the full information on how the system is going to perform in practice, especially when it has to counter too quick or too slow self-replication of RNA molecules. Another goal was to check how the model can be introduced into the RNA World in different ways. Inhibitors can act only on RNAs which replicate others or enzymes which perform other functions.

### 3.1. Spreadsheet Simulation

This is the simplest of all the simulations carried out, as it seeks to find an approximate solution to the differential equations presented in one of the previous sections. Its main purpose was to verify the analytical results, illustrate the efficiency of inhibitors and test how they react to external interference.

**Figure 2:**
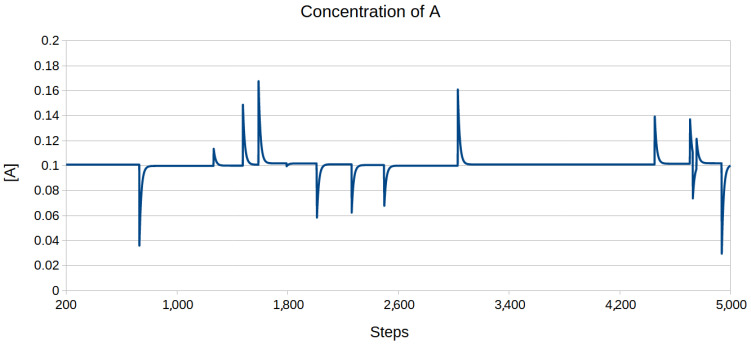
The concentration of *A* as a function of time. The system needed the first 200 steps to reach the equilibrium, so they were omitted. It is clearly visible that after each disturbance, the concentration always returned to its equilibrium value.

In order to solve Equations (31)–(35) numerically, they were discretised, with infinitesimal values dt, d[A],...being replaced with real numbers Δt, Δ[A],...Five thousand steps were simulated, all the parameters used are given in Table 1. The constants $k_{1}$, $k_{2}$, $k_{3}$, and $k_{4}$ were arbitrarily set to 1, while the rest of the parameters were chosen to give a relatively big capacity (β=63.76). By using Formula (62), one can calculate that the target concentration of *A* in this simulation is 0.1. Initially, all the molecules were uncomplexed ([XY]=0 and [AX]=0), so in order to test the system, it had to reach the equilibrium first, which took around two hundred steps. After the equilibrium had been reached, during each time step, the concentration of *A* could be modified with the probability of 0.2% by adding a random number from range [−0.075; 0.075] (uniform distribution). Figure 2 shows the changes in [A] over time. The inhibitors turned out to be very effective at countering any changes in the concentration of *A*, bringing it back to its equilibrium density of 0.1.

### 3.2. Efficiency Against a Predefined Noise Function

**Figure 3:**
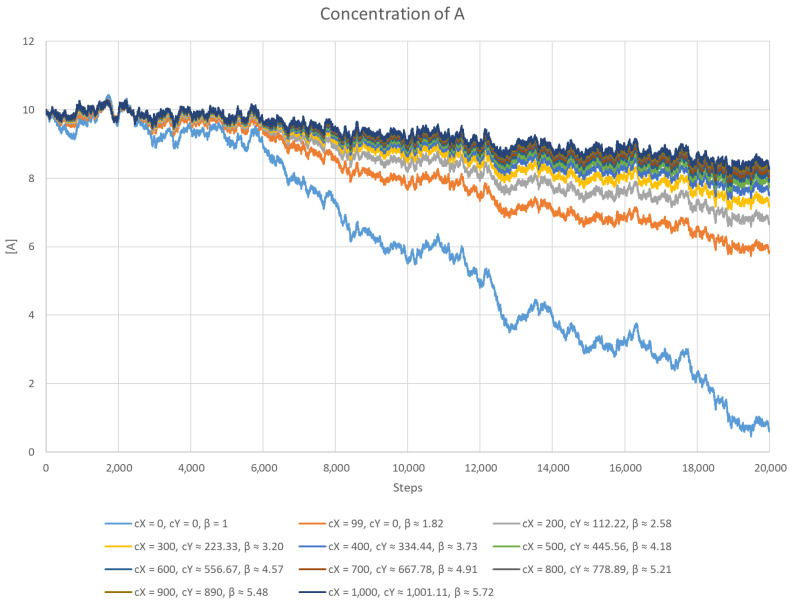
The concentration of *A* as a function of time with a falling noise function. The higher the concentration of the inhibitor, the better the result. Below, the values of the parameters are given: $c_{X}$ (the total concentration of the inhibitor), $c_{Y}$ (the total concentration of the anti-inhibitor), and β (the system capacity).

**Figure 4:**
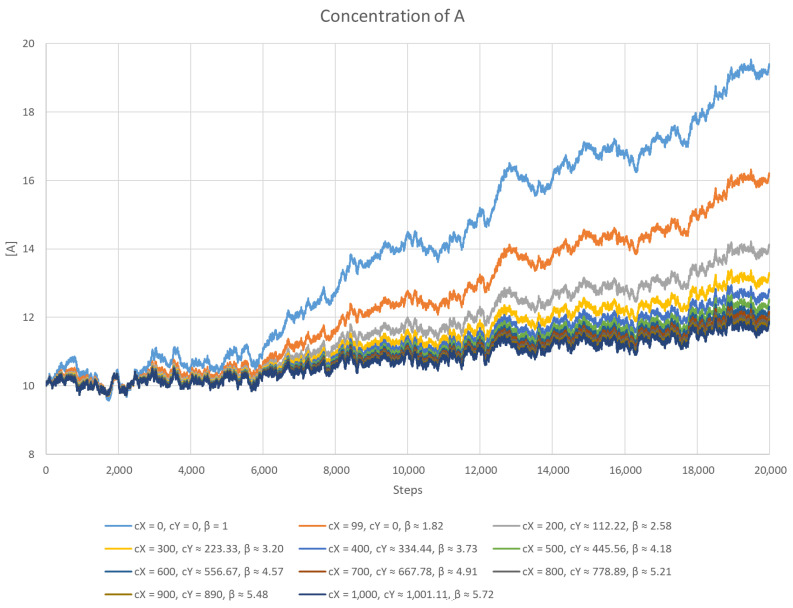
The concentration of *A* as a function of time with a rising noise function. The higher the concentration of the inhibitor, the better the result. Below, the values of the parameters are given: $c_{X}$ (the total concentration of the inhibitor), $c_{Y}$ (the total concentration of the anti-inhibitor), and β (the system capacity).

This scenario is very similar to the previous one and was created to test how well the system performs with different concentrations of the inhibitor. During the process, the equations were solved numerically by a program written in C++. A noise function was introduced, the sole purpose of which was to destabilise the system by changing the concentration of *A*. All the parameters were chosen arbitrarily. The equilibrium value of [A] was set to 10, while $c_{A}$ was equal to 100 (except for the case without inhibitors, where $c_{A}=\left[A\right]=10$). All the reaction rate constants were set to 1, and the length of the time step was 0.001. For a given value of $c_{X}$ (total inhibitor concentration), Equation (84) was used to calculate the corresponding value of $c_{Y}$ (total anti-inhibitor concentration), so that the equilibrium remained with [A]=10. The results are presented in Figure 3 and Figure 4. The case $c_{X}=99$ represents the simpler version of the model where $c_{Y}=0$. For each scenario, the system capacity β was calculated.

It is easy to notice that the simpler version of the model performs much worse than the one with anti-inhibitors present. Another observation is that the higher the concentration of the inhibitor, the better the stability of the system, which can better counter any changes in the concentration of *A*. It is important to note that this better performance is not affected by the concentration of [AX], since for all values of $c_{X}$ except 0, it was the same.

### 3.3. Inhibitors Working with Self-Replicating RNAs (Replicases)

One of the scenarios which had to be tested is how the inhibitors can manage the growing population of self-replicating RNA molecules. In this case, the equations were also simulated directly, but another modification was introduced instead of the noise function—the agent *A* was given the ability to self-replicate, that is, it always tried to gradually increase its numbers. It was achieved by modifying Equation (31) by adding two additional processes: replication (of rate $k_{RR}$) and decay (of rate $d_{R}$). The modified version can be written as follows:

(88) $$\frac{d[A]}{dt}=k_{RR}{\left[A\right]}^{2}-d_{R}\left[A\right]+k_{2}\left[AX\right]-k_{1}\left[A\right]\left[X\right]$$

The replication reaction can be written as

(89) $$2A\overset{k_{RR}}{\to }3A$$

**Figure 5:**
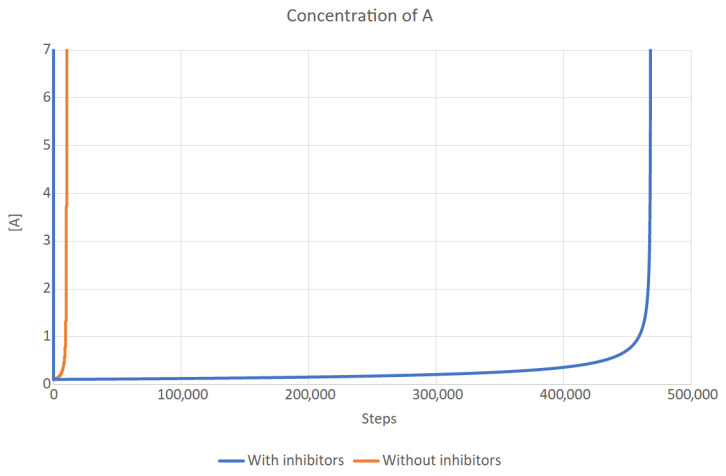
The concentrations of *A* over time with and without inhibitors. In the latter case, the population quickly grows out of control, while in the former, it remains stable for a long time, although it also explodes eventually.

All the parameters used in this simulation are presented in Table 2; they are identical to those in the spreadsheet simulation (with the equilibrium concentration of [A] equal to 0.1). Two scenarios were simulated: with and without inhibitors. The changes in [A] over time are shown in Figure 5. It is clearly visible that without any means of control (in this case, [A] was equal to 0.1 from the beginning), the population of replicases quickly explodes. While inhibitors cannot prevent this from occurring indefinitely, they can delay it by a huge amount of time. This means that they can make other control mechanisms more effective, by reducing the strain put on those mechanisms. This case is essential, as it clearly shows that like every system in nature, inhibitors have their limits.

### 3.4. Multi-Agent Simulation

The base for the algorithm was the simulation written in C# and presented in [49]. It was modified by adding four new types of agents:

1. Inhibitors;
2. Anti-inhibitors;
3. Inhibitor–anti-inhibitor complexes;
4. Inhibitor–parasite complexes.

Diffusion is handled in a similar way as for other molecules present in the simulation: all the four agents listed above diffuse freely, the first two with parameter *D*, while the latter two use $D^{\prime }$ instead. The inhibitors and anti-inhibitors do not decay, so their number (counting both the complexed and uncomplexed ones) remains constant during the entire simulation. The inhibitors can react with the anti-inhibitors and, depending on the scenario, replicases or parasites (playing the role of the molecule *A*) with the probability determined by parameters k1, k2, k3, and k4. These correspond to the constants in Equations (31)–(35), and each of them can be converted into the probability (for the time step of length Δt)

(90) $$p_{i}=1-e^{-k_{i}\Delta t}\;\mathrm{for}\;i=1,2,3,4$$

The formula above assumes that all reactions happen according to the Poisson distribution.

**Figure 6:**
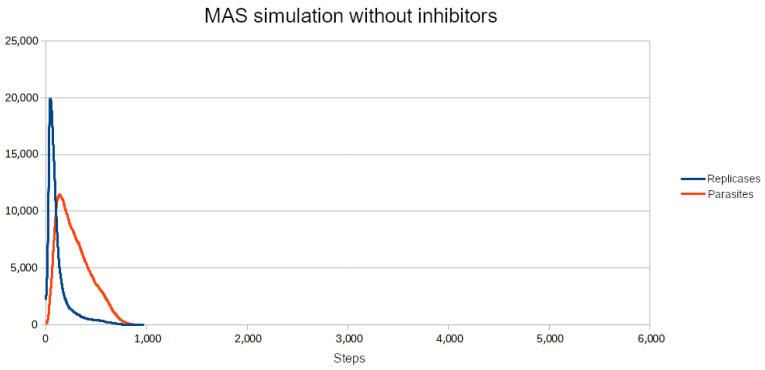
Without inhibitors, parasites dominated the system and caused the extinction of replicases and, in turn, the entire RNA population.

**Figure 7:**
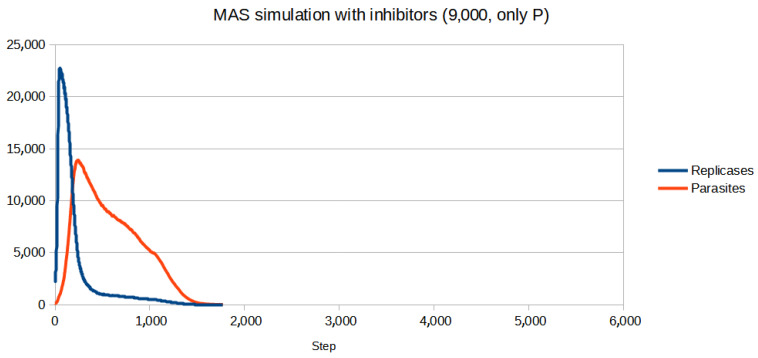
The concentrations of inhibitors and anti-inhibitors were not large enough to prevent the system from dying, but the extinction was staved off until almost 2000 steps had passed.

**Figure 8:**
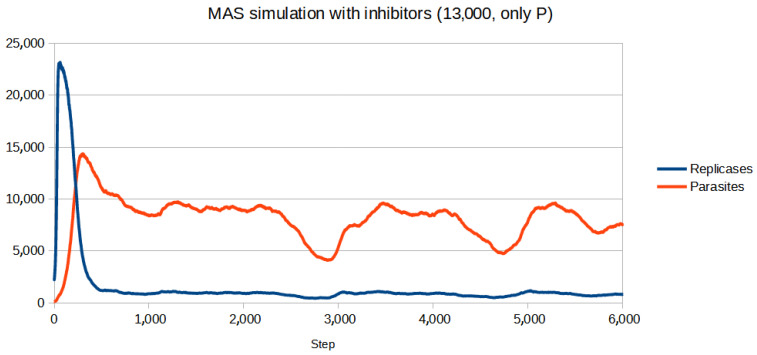
With the concentration close to the perfect one, the system was saved from extinction, although some fluctuations occurred.

**Figure 9:**
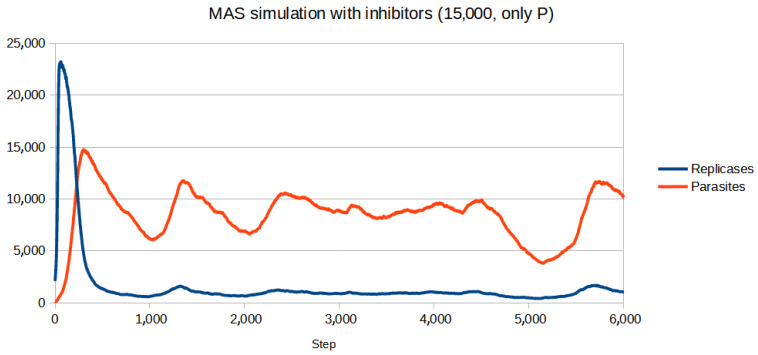
The high amount of inhibitors still allowed the system to survive and did not change the plot significantly.

**Figure 10:**
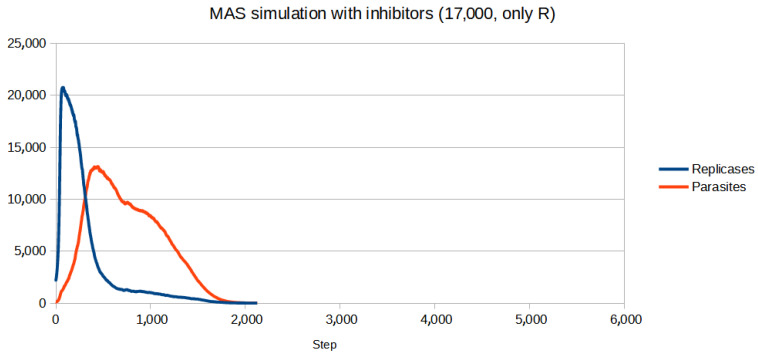
While 17,000 inhibitors allowed the system to exist when only parasites were blocked, it was not enough when replicases were controlled.

**Figure 11:**
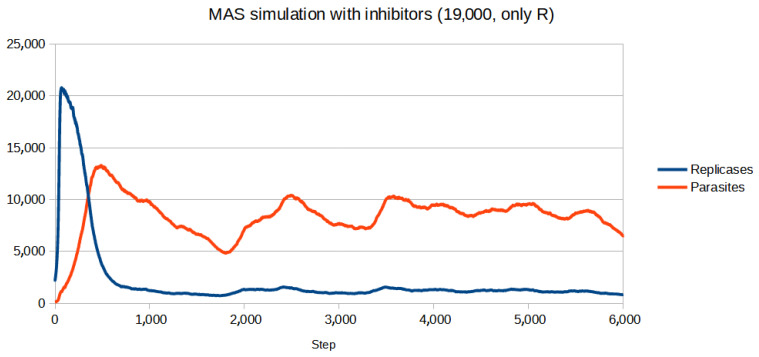
The first value which allowed the system to survive when only replicases were blocked was 19,000 of inhibitors. It was a significantly higher number than that of parasites.

To test if the inhibitors are effective in controlling the system, first, a set of parameters for which the population (without inhibitors) goes extinct was chosen. Then, the parameters of inhibitors had to be adjusted. Since for all four reaction constants equal to 1 (like in the previous simulations) inhibitors turned out to be too weak to be effective, they were increased. All the parameters are given in Table 3. The scenario without inhibitors is shown in Figure 6. Two versions of the simulation with inhibitors were carried out, where the number of inhibitors was always equal to the number of anti-inhibitors:

- Only parasites react with inhibitors (only P): The simulation was performed for different numbers of inhibitors ranging from 8000 to 15,000 with step 1000. The results of the three most representative cases are shown in Figure 7, Figure 8 and Figure 9.
- Only replicases react with inhibitors (only R): The number of necessary inhibitors turned out to be much bigger, so the simulation was carried out with 17,000, 18,000, 19,000, and 20,000 inhibitors. The results of two pivotal cases are presented in Figure 10 and Figure 11.

The introduction of inhibitors changes the outcome when their concentration is right. It is easy to notice fluctuations, which may be the result of a delay in the inhibitor system, since all the reactions take some time to occur. The most important result, however, is the fact that controlling the population by blocking replicases seems significantly harder than focusing solely on parasites. For replicases more inhibitors are required, even though the parasites outnumber the replicases for the majority of the simulation. It could be due to self-replication, which puts more strain on any controlling mechanisms being used.

## 4. Conclusions

The ability to maintain proper concentrations of enzymes and other active molecules is an important aspect of living things. Such a vital mechanism had to emerge at some point during evolution. In the RNA World, it could have given the newly forming compartments a serious advantage over competitors and, after aeons of constant improvement, evolved into the plethora of homeostasis processes we know today. The mechanism behind this had to be relatively simple at first if one assumes that it formed by chance. One of the possibilities is a system based on short RNA molecules, which could work according to the differential equation analysis proposed in this paper. This theory is strengthened by the fact that it does not require any additional molecules to have existed back in the RNA World. Various computer simulations presented here also showed its viability, by testing different aspects and behaviour in different scenarios with differing parameter sets. The proposed model can efficiently stabilise the levels of controlled molecules against interference and limit the growth of self-reproducing replicases. The introduction of inhibitors into the multi-agent system simulating the RNA World caused the entire system to be less extinction-prone. While this theory needs a confirmation in vitro besides the one in silico, it presents a promising direction for further research into the survivability of the first RNA populations in the RNA World.

While the mathematical analysis gave the proof that the system will tend to an equilibrium for every set of parameters, each simulation demonstrated an important aspect of the regulatory mechanism, taking a more practical approach. The spreadsheet was used to validate the mathematical results and to visualise how efficient the whole mechanism is for a high value of the system capacity, proving that in theory, inhibitors can be extremely effective. This was reinforced by testing the regulatory system against random noise, which further demonstrated that in a protocell environment with multiple processes going on in the background, the mechanism presented in the article can still function. Since one of the most important aspects of the RNA World is RNA replication, a proper scenario was tested, and the result is less optimistic, as ribozymes can eventually overpower inhibitors if given enough time and resources, meaning that in the RNA World, other means of control should have been present to effectively curtail uncontrolled molecule population growth. Last but not least, a scenario being the closest to reality was checked, using multi-agent simulation, which could model more processes going on in the primordial environment explicitly. As expected, the efficiency of inhibitors was much lower than in more controlled settings; however, they could change the outcome for some populations, which can be an argument corroborating the hypothesis that inhibitors gave an evolutionary advantage to the protocells in which they were present.

## Figures and Tables

**Table 1:** Parameters used in the spreadsheet simulation.

Parameter	Value
Δt	0.001
$k_{1}$	1
$k_{2}$	1
$k_{3}$	1
$k_{4}$	1
$c_{X}$	100
$c_{Y}$	24.45
$c_{A}$	7

**Table 2:** Parameters in the simulation with self-replicating molecules *A*.

Parameter	Value	Description
${\left[A\right]}_{0}$	7.0	Initial concentration of *A*
$c_{X}$	100	Initial (and total) concentration of *X*
$c_{Y}$	24.45	Initial (and total) concentration of *Y*
$k_{RR}$	1	Replication reaction rate
$d_{R}$	0.01	Decay rate
$k_{1}$	1	Constant $k_{1}$
$k_{2}$	1	Constant $k_{2}$
$k_{3}$	1	Constant $k_{3}$
$k_{4}$	1	Constant $k_{4}$
dt	0.001	Time step length

**Table 3:** Initial parameters in the multi-agent simulation.

Parameter	Default Value	Description
sizeX	1050	Simulation area width
sizeY	675	Simulation area height
init_R	2250	Initial number of replicases
init_P	112	Initial number of parasites
agent_size	3.0	Agent radius
$N_{max}$	4	Maximum number of neighbours
*d*	0.1	Decay rate
$a_{R}$	0.8	Replicase-to-replicase affinity
$a_{P}$	0.8	Parasite-to-replicase affinity
$l_{P}$	0.2	Parasite folding probability
*D*	400	Diffusion constant
$D^{\prime }$	400	Diffusion constant for complexes
δ	0.1	Parasite mutation rate
dt	1	Time step length
*K*	1	Replication constant
$k_{1}$	10	Constant $k_{1}$
$k_{2}$	5	Constant $k_{2}$
$k_{3}$	10	Constant $k_{3}$
$k_{4}$	5	Constant $k_{4}$

## Data Availability

Any data or materials that support the findings of this study can be made available by the corresponding authors upon request.
